# Tissue Residues and Pharmacokinetic/Pharmacodynamic Modeling of Tiamulin Against *Mycoplasma anatis* in Ducks

**DOI:** 10.3389/fvets.2020.603950

**Published:** 2020-11-27

**Authors:** Sara T. Elazab, Nahla S. Elshater, Yousreya H. Hashem, Seung-Chun Park, Walter H. Hsu

**Affiliations:** ^1^Department of Pharmacology, Faculty of Veterinary Medicine, Mansoura University, El-Mansoura, Egypt; ^2^Reference Laboratory for Veterinary Quality Control on Poultry Production, Animal Health Research Institute, Agriculture Research Center, Giza, Egypt; ^3^Mycoplasma Research Department, Animal Health Research Institute, Agriculture Research Center, Giza, Egypt; ^4^Laboratory of Veterinary Pharmacokinetics and Pharmacodynamics, College of Veterinary Medicine, Kyungpook National University, Daegu, South Korea; ^5^Department of Biomedical Sciences, College of Veterinary Medicine, Iowa State University, Ames, IA, United States

**Keywords:** tiamulin, HPLC, pharmacokinetics, pharmacodynamics, withdrawal time

## Abstract

The pharmacokinetics of tiamulin were studied in 2 groups of ducks (*n* = 6) after its oral administration at 2 different doses (30 and 60 mg/kg, respectively). Plasma concentrations of tiamulin were measured by high performance liquid chromatography at different time points up to 24 h post-administration. The maximum plasma concentrations were 0.77 and 2.32 μg/mL attained at 2 h (T_max_) for 30 and 60 mg/kg, respectively. The elimination half-lives for these 2 doses were 3.54 and 6.34 h, respectively. The minimum inhibitory concentration for tiamulin against *Mycoplasma anatis* (*M. anatis*) strain 1340 was determined to be 0.06 μg/mL. The proper oral dose of tiamulin against *M. anatis* in ducks was calculated to be 35 mg/kg/day using the pharmacokinetic/pharmacodynamic modeling. Tiamulin was administered orally (40 mg/kg/day) to 30 ducks for 3 successive days to determine its residues in edible tissues and its preslaughter withdrawal time. The highest tiamulin residues were detected in the liver, followed by the muscle, whereas lower concentrations were detected in the skin and fat. The estimated withdrawal periods of tiamulin were 6, 5, 3, and 3 days for liver, muscle, skin, and fat, respectively. Therefore, an oral dosage regimen of 35 mg/kg/day should be adequate for tiamulin against *M. anatis*. We recommend a preslaughter withdrawal period of 6 days when ducks are treated with 40 mg tiamulin/kg/day, orally, for 3 days.

## Introduction

Tiamulin was first isolated from the fungus *Pleurotusmutilis* in 1951 (1). It is available as a semisynthetic antibiotic which is a derivative of pleuromutilin. Tiamulin has high activity against Gram-positive bacteria as well as *Mycoplasma* spp. and *Leptospirae* (2–4). Tiamulin exhibits its bacteriostatic effect through blocking the 50S ribosomal subunit of bacteria, inhibiting peptidyl transferase (5). It is employed in veterinary medicine, particularly as a treatment for pneumonia, dysentery, gastrointestinal and mycoplasmal infections in swine and poultry (6–8). Tiamulin is also effective against mycoplasmal infection in ducks and geese (9).

The high lipid solubility of tiamulin accounts for its extensive distribution throughout the tissues of birds and mammals (10, 11). The pharmacokinetics of tiamulin have been studied in pigs (11, 12), piglets (13), goats, ewe, cows, calves (14), chickens and turkeys (10, 12, 15, 16). However, there are no data available on the pharmacokinetics of tiamulin in ducks.

*Mycoplasma anatis* affects predominantly ducks and eggs; the infection in ducks is usually associated with conjunctivitis, rhinitis, sinusitis, arthritis, growth retardation and reduced hatchability (17). The principal route of disease transmission is through eggs (vertical transmission) (18). Tiamulin has been utilized successfully as a treatment for mycoplasmosis in ducks and geese (9, 19, 20). It is crucial to optimize tiamulin dosage schedule to maximize clinical effectiveness, in addition to minimizing the development of drug resistance. Pharmacokinetic/pharmacodynamic (PK/PD) modeling points out the correlation between the pathogen, drug, and the treated animal, which in turn provides valuable information in the establishment of appropriate dose regimens for favorable clinical outcomes and for preventing the emergence of drug resistance (21, 22). To the best of our knowledge, the PK-PD integration of tiamulin against *M. anatis* has not been studied yet.

Avoiding antibiotic residues in edible products obtained from medicated animals is essential to ensure consumer's safety (23). The committee for Veterinary Medicinal Product (CVMP) of the European Union (EU) announced the Maximal Residual Limit (MRL) for tiamulin in tissues of chickens and turkeys to be 0.1 μg/g for muscles, skin, and fat, 1 and 0.3 μg/g for liver of chickens and turkeys, respectively (24). The tissue residue depletion of tiamulin has been reported in chickens (25), rabbits (26), and pigs (27). To the best of our knowledge, no reports are available concerning the depletion of tiamulin residues from tissues of ducks.

Therefore, the goals of this study were: (a) to investigate the pharmacokinetic behavior of tiamulin in healthy ducks after oral administration; (b) to study antimicrobial efficacy of tiamulin against *M. anatis*, (c) to calculate the rational dose of tiamulin against *M. anatis* in ducks based on the AUC24 h/MIC (the ratio of area under the concentration-time profile from 0–24 h: minimum concentration of tiamulin inhibiting the tested strain of *M. anatis*) for PK/PD modeling, and (d) to determine tiamulin residues in liver, muscle, skin and fat after treatment with our suggested clinical dose of tiamulin in ducks in order to estimate its preslaughter withdrawal period in ducks designated for human consumption to guarantee food safety.

## Materials and Methods

### Materials

Tiamulin hydrogen fumarate solution (Denagard®, 12.5%, Elanco Co., Basel, Switzerland) was utilized in this study. The tiamulin standard was purchased from Sigma Aldrich Co. (St. Louis, MO. USA). The high-performance liquid chromatography (HPLC) grade methanol, acetonitrile, potassium dihydrogen phosphate, hexane, ethyl acetate, sodium carbonate, ammonium carbonate, phosphate acid, and tartaric acid were obtained from Merck (Darmstadt, Germany). Purified water was produced by Milli–Q system (Waters Corp., Milford, MA. USA). The *M. anatis* standard strain 1340 was supplied by Animal Health Research Institute, Giza, Egypt. *M. anatis* medium base was made according to a published method (28).

### Animals

Forty-two healthy Muscovy ducks (10 ± 1 weeks, 2.0–2.5 kg, male: female, 1:1) were procured from Faculty of Agriculture, Mansoura University, Egypt. They were housed in adjusted climate at 23–27°C and 45–65% relative humidity. The ducks were acclimated for 14 days before the start of the experiment. Drug-free feed and water were consumed *ad libitum*. Approval for the animal experiments was secured from the Animal Ethics Committee at the Faculty of Veterinary Medicine, Mansoura University (Approval No. R/44).

### Experimental Design

In this research, two separate experiments were executed; in the 1st experiment, 12 ducks were randomly divided into two groups (*n* = 6, 3 males and 3 females). Group 1 and 2 were gavaged with tiamulin directly into the crop via a plastic tube linked to a syringe with a single dose of tiamulin at 30 and 60 mg/kg, respectively. These doses were chosen based on the suggested dosage of 30–60 mg/kg, orally, for 3–5 days (6). Food was withheld for 6 h before and after tiamulin administration, while water was consumed *ad libitum*. Blood samples of 1 mL were taken from a wing vein into EDTA tubes at time 0 (prior to tiamulin administration), and 0.5, 1, 2, 4, 6, 8, 10, 12, and 24 h post-tiamulin administration. Blood samples were centrifuged at 1,257 × *g* for 10 min to yield plasma which were immediately stored at −20°C until analysis.

In the 2nd experiment, 30 ducks were administered tiamulin orally via gavage at 40 mg/kg daily for 3 successive days (6, 16). After the 3rd day of treatment, 6 ducks each were slaughtered on 1st, 3rd, 5th, 7th, and 9th day after the tiamulin dosing. Tissue samples (liver, breast muscle, abdominal skin and subcutaneous fat) were collected from each duck and were frozen at −20°C until analysis.

### Analysis of Tiamulin in Plasma Samples

#### Extraction of Tiamulin From Plasma Samples

The preparation of plasma samples was performed based on a former published technique (29). Briefly, 0.5 mL plasma sample was mixed with 2.5 mL 1% aqueous solution sodium carbonate and then 2,5 hexane-ethyl acetate (3/1 v/v) was added for extraction. After shaking, the mixture was centrifuged at 2,360 × g for 20 min. Then, 1 ml of supernatant was evaporated under nitrogen stream. After that, 0.1% buffered aqueous solution tartaric acid (0.3 mL) was added to redissolve the residue. Ten microliters of the sample was injected into the HPLC system.

#### Chromatographic Conditions

Tiamulin concentrations in the standards and plasma samples were determined following a published HPLC method with few modifications (29). The HPLC Agilent Series 1200 quaternary gradient pump, Series 1200 Autosampler, Series 1200 UV VIS detector set at 210 nm, and HPLC 2D Chemstation software (Hewlett-Packard, Les Ulis, France) were operated. Chromatographic analysis was performed utilizing a Phenomenex C18 column (5 μm, 150 mm × 4.6 mm). Acetonitrile and potassium dihydrogen phosphate (pH = 2.8) (65:35, v/v) were used as a mobile phase under isocratic conditions. The flow rate was 1.5 mL/min. The retention time was 1.2 min. The method used for HPLC analysis was revalidated as described by a European Medicines Agency protocol (30) utilizing duck plasma (Table 1). The calibration curve was established by depicting peak area vs. concentration of tiamulin using the data from 9 concentrations (ranging 0.01–5 μg/mL plasma). A linear correlation was identified in the calibration curve in the range of 0.01–5 μg/mL. The lower limits of detection and quantification of tiamulin were 0.003 and 0.01 μg/mL, respectively. The intra- and inter-day precision and accuracy of the assay was indicated in Table 1.

**Table 1:** Validation parameters of the HPLC method used for analysis of plasma samples.

**Matrix**	**Average recovery (%)**	**Intra-day RSD (%)**	**Inter-day RSD (%)**	**LOD(μg/mL)**	**LOQ (μg/mL)**
Plasma	98.50 ± 1.34	0.48	0.37	0.003	0.01

*Data for recovery are presented as mean ± SEM. Intra-day RSD and Inter-day RSD % (n = 6, 0.025 μg/mL). Average recovery % (using spiked concentrations in the range of 0.01–5 μg/mL in triplicate analysis)*.

### Analysis of Tiamulin in Tissue Samples

#### Extraction of Tiamulin From Tissue Samples

Tissue samples were prepared as reported (31). In brief, tissue samples were mixed with acetonitrile, purified by liquid partition separation. At last extraction was performed using n-hexane. Then the extract was concentrated and eluted via solid phase extraction cartridge column (Bond Elut C18, 3 mL/500 mg, Varian Company, Palo Alto, CA, USA) for HPLC analysis.

#### Chromatographic Conditions

The analysis of tiamulin in tissue samples (liver, muscle, skin, and fat) was undertaken utilizing a published method (31). Briefly, The HPLC Agilent Series 1200 quaternary gradient pump, Series 1200 Autosampler, Series 1200 UV VIS detector set at 210 nm, and HPLC 2D Chemstation software (Hewlett-Packard, Les Ulis, France) were employed. The HPLC column was a Phenomenex C18 (5 μm, 250 mm × 4.6 mm). The mobile phase comprised a mixture of 80% acetonitrile and 1% ammonium carbonate (90:10, v/v) at a flow rate of 1.0 mL/min. The retention time was 9.3 min. The calibration curve of peak area vs. tiamulin concentration was plotted with data from 8 concentrations [0.025–5 μg/g tissue (liver, muscle, skin, and fat)]. The analytical technique was validated using duck tissues (Table 2). Linearity of this technique was observed (*R*^2^ > 0.99) in the standard curve. The lower limits of detection and quantification of tiamulin were 0.008 and 0.025 μg/g, respectively.

**Table 2:** Validation parameters of analytical HPLC technique utilized for investigation of the tissue samples.

**Matrix**	**Average recovery (%)**	**Intra-day RSD (%)**	**Inter-day RSD (%)**	**LOD (μg/mL)**	**LOQ (μg/mL)**
Liver	98.30 ± 1.70	2.90	2.20	0.025
Muscle	100.70 ± 0.92	2.37	3.78	0.008	0.025
Skin	101.90 ± 0.81	3.40	4.20	0.008	0.025
Fat	101.80 ± 1.31	4.10	3.50	0.008	0.025

*Data for recovery are shown as mean ± SEM. Intra-day RSD and Inter-day RSD % (n = 6, 0.05 μg/g). Average recovery % (using spiked concentrations in the range of 0.025–5 μg/g in triplicate analysis)*.

#### Evaluation of Minimum Inhibitory Concentration (MIC) of Tiamulin Against *M. anatis*

The determination of MIC of tiamulin against *M. anatis* strain 1340 was performed as reported (32). In brief, *M. anatis* titer of 10^7^ CFU/mL in the mycoplasma medium was prepared. A series of tiamulin standard concentrations were made in the range of 0.03125–32 μg/mL using *M. anatis* culture medium as the diluent (1:1 dilution). The MIC was identified as the lowest concentration of tiamulin where no change in the color of culture medium was noticed.

#### *In vitro* Time-Killing Curve

The *in vitro* time-killing studies were conducted according to a reported technique (32). Different concentrations of tiamulin which were multiples of MIC were produced (0, 0.5, 1, 2, 4, 8, 16, 32, and 64 MIC). The *M. anatis* titer with inoculum size of 10^7^ CFU/mL was made by adding 0.4 mL of the suspension in exponential phase to 7-mL penicillin bottles; each bottle had 0.1 mL of tiamulin solution and 3.5 mL blank medium. The cultures were kept in the incubator at 37°C with 5% CO_2_ for 48 h. Aliquots of 100 μl from each culture were collected at 0, 2, 12, 15, 18, 24, 26, 36, 38, 40, and 48 h. The viable counts of *M. anatis* were estimated. Investigation for each concentration was performed in triplicate. The mean log_10_ (CFU/mL) values (*n* = 3) were plotted against time (h) with different concentrations of tiamulin for construction of the *in vitro* time-killing curve.

#### *Ex vivo* Time-Killing Curve

Plasma samples harvested from healthy ducks which had been given tiamulin by gavage at 60 mg/kg were used for the time killing experiments. The assessment of the anti-mycoplasmal effect of tiamulin was conducted using plasma samples collected at the following time points: 0, 0.5, 1, 2, 4, 6, 8, 10, 12, and 24 h after tiamulin administration. The viable counting of *M. anatis* was done using the aforementioned method for the *in vitro* time killing studies. For depicting the time-killing curve, the mean log_10_ (CFU/mL) values (*n* = 6) were plotted vs. time (h) with different plasma samples taken from ducks which had received tiamulin at various time points.

#### Determination of Pharmacokinetics Profile and Preslaughter Withdrawal Time

The pharmacokinetic profile of tiamulin was investigated utilizing the non-compartmental approach [WinNonlin 8.3 software (Certara, USA)] as reported (15, 16). The values of the highest plasma concentration (C_max_) and the time required to attain C_max_ (T_max_) were recorded from the plasma concentration-time plot. The area under the plasma concentration-time curve (AUC_0−∞_) was computed using the linear-log trapezoidal method. The elimination half-life (T_1/2_λz) was calculated using the equation T_1/2_λz = 0.693/λz.

The withdrawal time (WT) was calculated by applying WT 1.4 program which was established in Germany and approved by the CVMP of the EU. It was estimated utilizing the statistical approach (95% tolerance limit and 95% confidence interval) based on the EU MRL for tiamulin in chicken tissues, which were announced by the CVMP to be 0.1 μg/g for muscles, skin, and fat, and 1 μg/g for liver, respectively (24).

#### PK/PD Modeling and Dose Determination

The value of the area under the concentration-time profile over 24 h (AUC_24h_) for the *ex vivo* time killing curve was computed by multiplying each tiamulin concentration measured after the oral administration at 60 mg/kg by 24 h (incubation period). Subsequently, the AUC_24h_ value was divided by the MIC to estimate the AUC_24h_/MIC (33–35).

The log_10_ difference between *M. anatis* count (CFU/mL) after 24 h incubation and the initial count was correlated with the AUC24 h/MIC ratio using the Sigmoidal inhibitory E_max_ model as illustrated by the equation:

$$\begin{array}{l} \text{E}={\text{E}}_{\text{0}}-(({\text{E}}_{\text{max}}\times {\text{C}}^{\gamma })/({\text{C}}^{\gamma }+{\text{EC}}_{50}^{\gamma })) \end{array}$$

In which E reflects the antimycoplasmal effect identified as the variation between *M. anatis* count (log_10_ CFU/mL) in the plasma sample after 24 h of incubation and the baseline log_10_ CFU/mL (the growth without tiamulin); E_max_ elucidates the highest antimycoplasmal activity; E_0_ represents the difference in *M. anatis* count in the control sample (without tiamulin) between 0 and 24 h of incubation; EC_50_ is the AUC/MIC ratio showing a 50% decline in *M. anatis* counts from the original count; C refers to the AUC/MIC in the effect compartment; γ is the Hill coefficient which describes the steepness of the AUC_24h_/MIC effect curve. These PD parameters were computed with WinNonlin 8.3 software (Certara, USA).

The antimycoplasmal activity of tiamulin was evaluated by calculating the AUC/MIC desired for bacteriostatic action (no variation in bacterial count after 24 h incubation, *E* = 0), and bactericidal action (99 and 99.9% decrease in bacterial count, *E* = −2 and *E* = −3, respectively) by utilizing the sigmoid E_max_ model.

Using the PK/PD modeling, the optimal daily dose for tiamulin in ducks was calculated based on this equation (36):

$$\begin{array}{l} \text{Dose}\;\left(per\;day\right)=\frac{\left(\frac{AUC_{24}}{MIC}\right)breakpoint\;\times \;MICx\;Clearnace\;\left(per\;hour\right)}{fu\;\times F} \end{array}$$

Where AUC_24h_/MIC indicates the AUC _24h_/MIC ratio for appropriate efficacy; MIC refers to the minimum inhibitory concentration; CL represents the clearance (L/h/kg); F reflects the bioavailability; *fu* is the free portion of tiamulin in plasma.

#### Statistical Analysis

Data are expressed as mean ± SEM. Normality of the data was examined by using Shapiro-Wilk test. Mann-Whitney test was used to compare the major pharmacokinetic parameters for the two different doses used (30 and 60 mg/kg). The counts of *M. anatis* of the time killing curves at 48 h were compared using one-way analysis of variance (ANOVA), followed by the Tukey's mean comparison test. Moreover, the residue levels of tiamulin were compared in various tissues using ANOVA, followed by the Tukey's mean comparison test. *P* < 0.05 was regarded as statistically significant. Statistical investigation was undertaken utilizing Statistical Package for Social Science (SPSS), version 20 (SPSS Inc., Chicago, IL, USA) for windows.

## Results

### Tiamulin Pharmacokinetics

**Figure 1:**
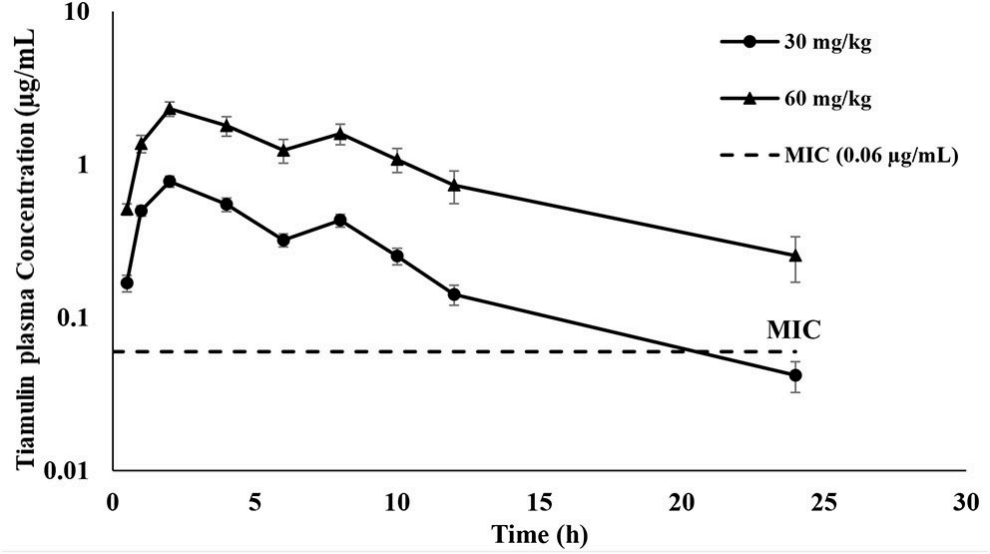
Plasma concentration-time curves of tiamulin in ducks following oral administration at 30 and 60 mg/kg. Values are shown as mean ± SEM (*n* = 6).

The plasma concentration-time curves of tiamulin following a single oral administration by gavage at 30 mg/kg and 60 mg/kg, respectively, are presented on a semilogarithmic graph in Figure 1. Tiamulin was detectable (≥3 ng/mL) in plasma up to 24 h after oral administration of two different doses of tiamulin. The main PK parameters of tiamulin are listed in Table 3. Statistical comparison of the parameters between the 2 doses showed no significant difference in the T_max_, T_1/2_λz, MRT, Vz_F_obs, and Cl_F_obs. Whereas, the differences in C_max_, AUC_0−last_, and AUC_0−∞_ were significant (*p* < 0.05) as their values increased in a dose-dependent manner.

**Table 3:** Pharmacokinetic parameters of tiamulin in ducks after oral administrations at 30 and 60 mg/kg BW.

**Parameters**	**Tiamulin at 30 mg/kg**	**Tiamulin at 60 mg/kg**
C_max_ (μg/mL)	0.77 ± 0.06	2.32 ± 0.25^*^
T_max_ (h)	2.00 ± 0.00	2.00 ± 0.00
λz (1/h)	0.24 ± 0.05	0.12 ± 0.02
T_1/2_ λz (h)	3.54 ± 0.68	6.34 ± 0.89
AUC_0−last_ (μg*h/mL)	5.55 ± 0.65	22.83 ± 3.98^*^
AUC_0−∞_ (μg*h/mL)	5.85 ± 0.68	25.69 ±4.94^*^
Vz_F_obs (L/kg)	25.78 ± 3.06	23.93 ± 3.12
Cl_F_obs (L/h/kg)	5.59 ± 0.83	2.61 ± 0.88
MRT (h)	5.95 ± 0.47	7.64 ± 0.45

*C_max_, maximum plasma concentration; T_max_, Time to the highest concentration; λz, the first order rate constant; T_1/2_ λz, elimination half-life; AUC_0−last_, area under the plasma concentration against time plot from 0 to last time; AUC_0−∞_, Area under the plasma concentration-time curve from 0 to infinite; V_z_obs_, volume of distribution scaled by bioavailability; Cl_F_obs, clearance divided by bioavailability; MRT, Mean residence time; Data are presented as mean ± SEM (n = 6)*.

* *p < 0.05*.

### Tiamulin PD

The MIC of tiamulin for *M. anatis* strain 1340 was estimated as 0.06 μg/mL. No growth of *M. anatis* was noted in the sterilizing control and end-point control, whereas *M. anatis* growth was observed in the positive control.

**Figure 2:**
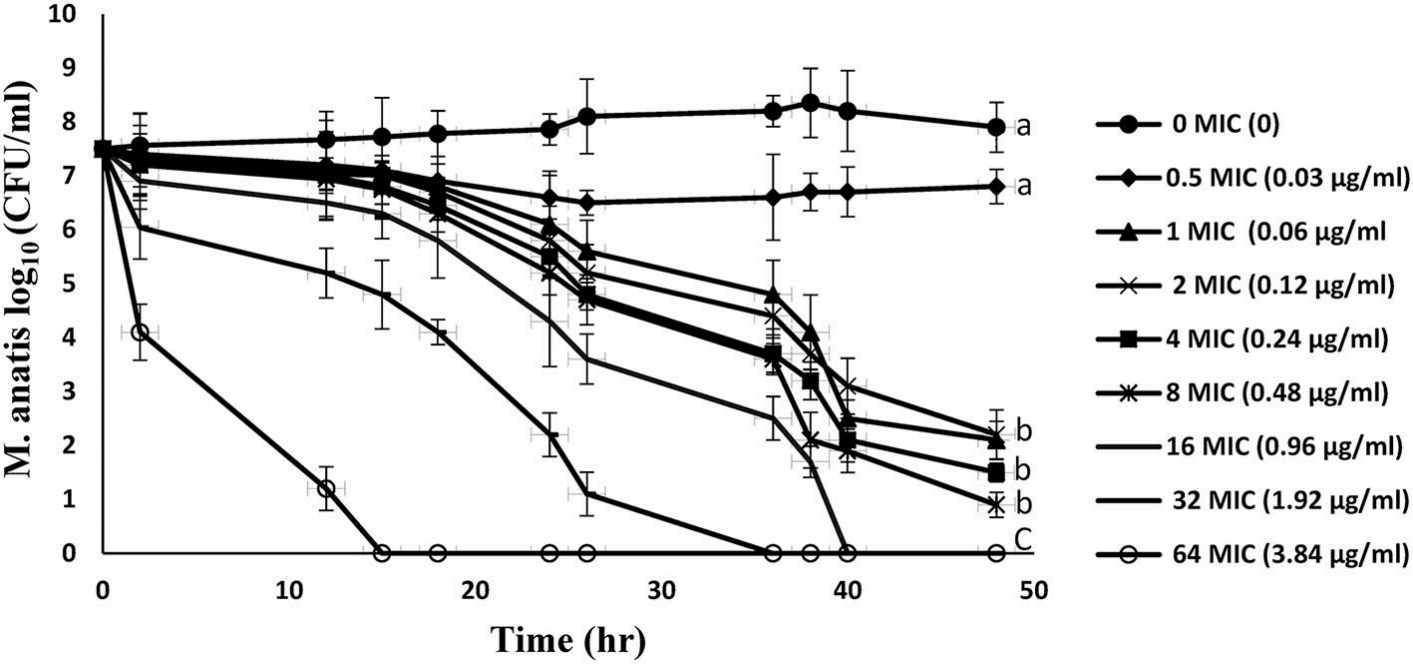
*In vitro* time-kill curves for tiamulin concentrations in the range of 0–64 MIC against *M. anatis* in the growth medium. Values are presented as mean ± SEM (*n* = 3).

Figure 2 represents the *in vitro* time-kill curves for 8 multiples of MIC (0.5–64 MIC) of tiamulin against *M. anatis* strain 1340 in artificial medium over a period of 48 h. These curves demonstrated a time-dependent effect of tiamulin vs. *M. antis* strain 1340, as the rate of *M. anatis* growth inhibition increased in response to the increment of the exposure time of *M. anatis* to tiamulin. Furthermore, as the tiamulin concentration increased (1–64 MIC), the antimycoplasmal effect increased since 64 MIC yielded the maximal killing action.

**Figure 3:**
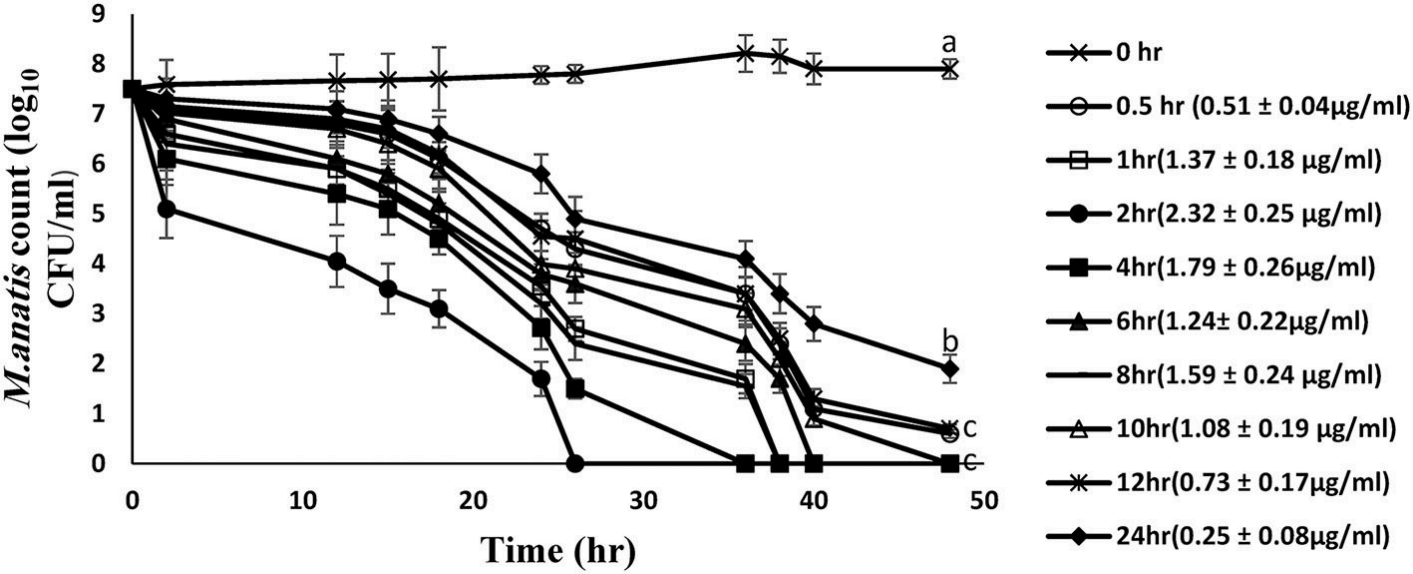
*Ex vivo* inhibition of *M. anatis* growth in plasma after oral administration of tiamulin (sampling times of 0, 0.5, 1, 2, 4, 6, 8, 10, 12, and 24 h). Values are expressed as mean ± SEM. (*n* = 6). Values (μg/mL) in brackets are the corresponding tiamulin concentration in the samples at different time points expressed as mean ± SEM.

The time-kill curves for tiamulin in duck plasma against *M. anatis* strain 1340 were elucidated at 8 time points using samples harvested following oral dosing of tiamulin at 60 mg/kg (Figure 3). Plasma samples collected between 0.5 and 12 h post-tiamulin administration exhibited mycoplasmocidal action (≥3-log_10_ CFU/mL) after 24 h of incubation. Slight inhibition of *Mycoplasma* growth (2-log_10_ CFU/mL reduction) was observed in 24 h samples. No *M. anatis* growth was detected after 48 h of incubation for samples collected between 1 and 10 h.

### PK/PD Modeling and Dose Determination

**Figure 4:**
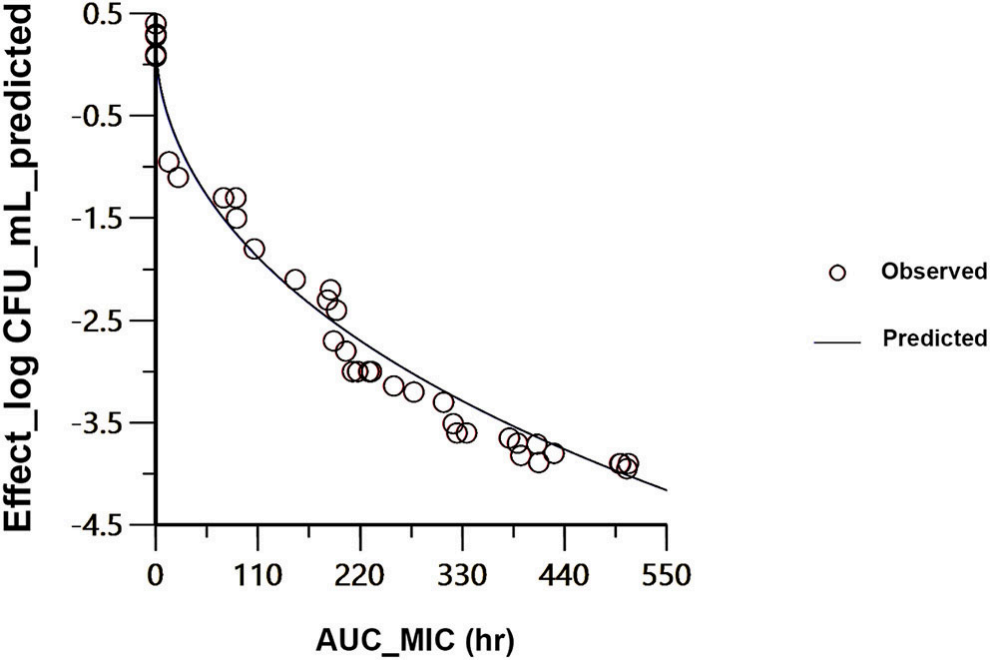
Sigmoidal E_max_ correlation for the mycoplasma count (Log_10_ CFU/ mL) vs. AUC_24h_/MIC for *M. anatis* in plasma of ducks. The plot reflects the line of predicted values, based on the sigmoid E_max_ equation, and the circles point out the values of the individual ducks.

The findings from PK/PD integration of time-killing curve are presented in Table 4 and Figure 4. The AUC_24h_/MIC required to produce mycoplasmastasis (no alteration in the number of mycoplasma, *E* = 0), mycoplasmocidal (99% decline in mycoplasma count, *E* = −2), and mycoplasmocidal (99.9% decline in mycoplasma count, *E* = −3) were 6.75, 122.2 and 251.9 h, respectively.

**Table 4:** Parameters of PK/PD modeling from *ex vivo* studies following oral administration of tiamulin at 60 mg/kg in ducks.

**Parameter**	**Units**	**Mean ± SEM**
E_max_	CFU/mL	6.00 ± 1.42
EC_50_	h	278.66 ± 122.55
E_0_	CFU/Ml	−0.16 ± 0.19
E_max_-E_0_	CFU/mL	6.17 ±1.62
AUC _24h_/MIC for bacteriostatic effect (*E* = 0)	h	6.75
AUC _24h_/MIC for bactericidal effect (*E* = −2)	h	122.2
AUC _24h_/MIC for bactericidal effect (*E* = −3)	h	251.9

*E_max_, is the maximum difference n log_10_ of bacterial number of sample incubated with tiamulin; EC_50_, is the PK/PD parameter of the drug that produces half of the maximal antibacterial effect; E_0_, the difference after 24 h of incubation in log_10_ of number of bacteria in control samples (without tiamulin); AUC_24h_/MIC, values required to achieve bacteriostatic effect and bactericidal effect*.

Based on the estimated AUC_24h_/MIC from PK/PD modeling, MIC of *M. anatis* (0.06 μg/ml) obtained in this study, CL_F_obs (clearance divided by bioavailability after oral administration of tiamulin at 60 mg/kg; 2.61 L/h/kg) which reflects the unknown bioavailability (F), and the free fraction of tiamulin of 0.55 (10), the dose of tiamulin needed per 24 h for bacteriostatic, bacteriocidal action (99% decline in mycoplasma count, *E* =-2), and bacteriocidal action (99.9% decline in mycoplasma count, *E* = −3) against *M. anatis* in ducks were determined to be 2 mg/kg, 35 mg/kg/day, and 72 mg/kg, respectively.

### Tissue Residues and Preslaughter Withdrawal Time of Tiamulin

**Figure 5:**
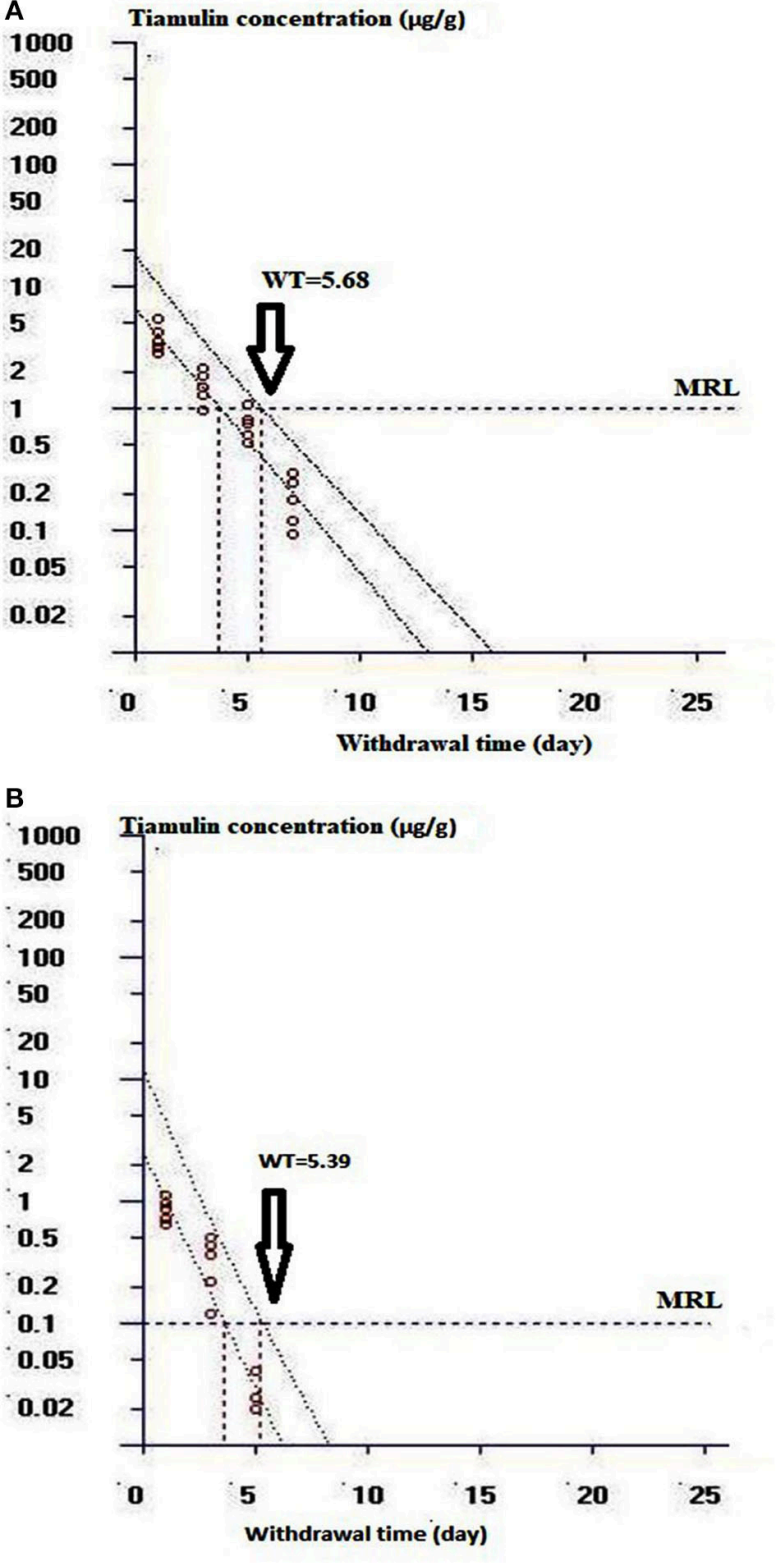
Curve of the withdrawal period estimation at the time when the one-sided 95% upper tolerance limit was below the EU MRL of 1 μg/g for liver and 0.1 μg/g for muscle. **(A)** Shows the withdrawal time determination for duck liver. **(B)** Shows the withdrawal time evaluation for duck muscle.

Table 5 illustrates the residue concentrations of tiamulin in duck tissues (liver, muscle, skin, and fat) after oral dosing at 40 mg/kg/day for 3 successive days. The highest tiamulin concentration was detected in the liver, followed by the muscle, whereas lowest concentrations were detected in the skin and fat. Furthermore, the present study showed that tiamulin was detected in the skin and fat up to the 3rd day post-dosing and up to the 5th and 7th day after the end of the treatment in muscle and liver, respectively. The withdrawal time was 5.7 days for liver (approximated to 6 days), and 5.4 days for muscle (approximated to 5 days) as elucidated in Figures 5A,B. Since the residues of tiamulin in skin and fat on the 3rd day after last dose were lower than EU MRL (0.1 μg/g), hence the 3-day withdrawal time for the skin and fat is suggested.

**Table 5:** Concentrations (μg/g) of tiamulin in duck tissues (liver, muscle, skin, and fat) following its oral administration at 40 mg/kg for 3 consecutive days.

**Time after the last dose (days)**	**Liver**	**Muscle**	**Skin**	**Fat**
1	3.62 ± 0.29^a^	0.89 ± 0.10^b^	0.35 ± 0.06^c^	0.28 ± 0.08^c^
3	1.48 ± 0.19^a^	0.31 ± 0.06^b^	0.08 ± 0.02^b^	0.05 ± 0.01^b^
5	0.73 ± 0.09^a^	0.04± 0.01^b^	ND	ND
7	0.17 ± 0.03	ND	ND	ND
9	ND	ND	ND	ND

*Data are presented as mean ± SEM (n = 6). Means in the same row with different superscript letters are statistically significant at p < 0.05. ND, not detectable (<0.025 μg/g)*.

## Discussion

This study was the first research about PK of tiamulin in ducks. The findings of this study revealed that tiamulin, when administered orally to ducks, was rapidly absorbed with C_max_ values [0.77 and 2.32 μg/mL attained at 2 h (T_max_) for 30 and 60 mg/kg, respectively] and these values were similar to those reported for dogs, goats, and ewes, which were injected with tiamulin intramuscularly (i.m.) at 10 mg/kg (0.60, 0.56, and 0.64 μg/mL, respectively) (14, 37), pig administered tiamulin orally at 10 mg/kg (0.69 μg/mL) (13), chickens received tiamulin orally at 40 mg/kg (0.73 μg/mL reached at 1.5 h) (16), and for chickens injected with tiamulin i.m. at 5 mg/kg (2.05 μg/mL) and 40 mg/kg (8.8 μg/mL) (15). These results suggested that the oral absorption of tiamulin is potentially affected by intestinal factors. Moreover, the present study showed a second peak at 8 h post-tiamulin administration with the two different doses. This is likely due to the enterohepatic recycling of tiamulin during biliary elimination (15, 16).

In addition, following oral administration of tiamulin at 30 mg/kg, the AUC extrapolated to infinity was 5.85 μg*h/mL. This value was comparable to that reported for chickens (5.18 μg*h/mL) (16).

Furthermore, our data revealed that after a single oral administration of tiamulin at 2 different doses, the T_1/2_λz (3.54 and 6.34 h) were short and these results suggested a rapid elimination of tiamulin in ducks. One potential reason for this short T_1/2_λz is the rapid clearance rate (2.61 and 5.59 L/h/kg, respectively, after the 2 doses). The relationship between the T_1/2_λz and Cl was elucidated by the following equation (37): T_1/2_λz= $\frac{0.693xVz}{Cl}$. These results were consistent with the findings in chickens (4.23 h) (16), dogs (2.23 h) (38), and pigs (2.14 h) (13). Additionally, the MRT (5.95 and 7.64 h for 30 and 60 mg/kg, respectively) observed in this study were comparable to the MRT of 7.1 h found in chickens treated with oral dose of tiamulin at 40 mg/kg (16). The Vz_F_obs for the 2 doses in ducks were 25.78 and 23.93 L/kg for 30 and 60 mg/kg, respectively. To the best of our knowledge, limited data are available about Vz of tiamulin in other species. The Cl_F_obs of tiamulin reported in this study were 5.59 and 2.61 L/h/kg for 30 and 60 mg/kg tiamulin doses, respectively. These values were nearly similar to the CL_F of 4.6 L/h/kg reported in *Mycoplasma gallisepticum* infected chickens injected i.m. with 40 mg/kg (15). In contrast, they were much lower than that reported in healthy chickens administered tiamulin at 40 mg/kg through the crop, drinking water, and feed (30.3, 26.4, and 14.7 L/h/kg, respectively) (16). However, these CL_F_obs values reported in chickens by Vinothini et al. (16) were much higher than that revealed in other species; for instance, in dogs the CL_F value was 1.36 L/h/kg after i.m. injection of tiamulin at 10 mg/kg (38). The interspecies variation in plasma clearance points out the differences in the relative importance of the multiple possible routes of drug excretion (liver and non-liver metabolism vs. biliary elimination vs. renal elimination) and the ability for any given method of elimination (39).

Moreover, in this study, tiamulin showed a time- and concentration-dependent effect against *M. anatis* in the *in vitro* time killing study. The antimycoplasmal action was augmented by the increment of drug concentration or the time of exposure. These results were in accordance with the work by Novak and Schlaes (7) in which the effects of most pleuromutilins were predominantly time-dependent. Moreover, Xiao et al. (40) reported that valnemulin was concentration-dependent against *M. gallisepticum*. It has been reported that the time- or concentration-dependent action of antibiotics may alter under various conditions (40).

The PK/PD index of pleuromutilin antibiotics is AUC_24_/MIC (41). This work elucidated that the MIC of tiamulin against *M. anatis* strain 1340 was 0.06 μg/mL. Using this MIC value, and the estimated AUC_24h_/MIC for mycoplasmacidal action (122.2 h), the calculated daily dosage of tiamulin to produce mycoplasmpcidal effect (2 log_10_ equivalent reduction in mycoplasma count) against *M. antis* in ducks would be 35 mg/kg/day. This dose was in the range of the recommended oral doses in poultry (30–60 mg/kg) (6).

In the present study, tiamulin showed a mycoplasmocidal effect. In the same line, valnemulin, a member of pleuromutilins, exhibited action against *M. gallisepticum* (15). On the contrary, Burch and Alvarz revealed that tiamulin did not achieve a mycoplasmocidal action against *M. gallisepticum* (42). This discrepancy may be due to the difference in the *Mycoplasma* species and/or the variation in the susceptibility to the drug. Xiao et al. reported that the bacteriostatic and bactericidal features may be varied for the same drug against different microorganisms (40).

The depletion of tiamulin residues from ducks' edible tissues was investigated after its administration at 40 mg/kg once daily for 3 successive days, as the recommended oral doses in poultry is 30–60 mg/kg bw for 3–5 days (6).

Based on the calculated withdrawal time of tiamulin in this study for liver, muscle, skin, and fat, it would be reasonable to suggest a preslaughter withdrawal time of 6 days after oral administration of 40 mg/kg tiamulin to ascertain a level of tiamulin lower than the MRL in duck tissues before being consumed by humans to ensure food safety. These results are in agreement with the report that after i.m. administration of tiamulin to pigs at 12 mg/kg once daily for 5 days, the most convenient preslaughter withdrawal period was 6 days (27). However, the withdrawal time calculated in the present study (6 days) was higher than that reported for chickens of 3–4 days (25, 43).

The tiamulin dose calculation was performed using the data derived from a single strain of *M. anatis*. However, it would be better to determine the dose according to estimates of MIC90 (the 90th percentile of MIC distribution) values to accommodate the potential needs of the entire patient population. To the best of our knowledge, limited data are available about MIC distribution for tiamulin against *M. anatis*, the breakpoint and the epidemiological cut-off value (ECOFF) due to the difficulty of culturing *M. antis* and the long doubling time. Hence, further studies are needed to validate the calculated dosage in clinical circumstances to confirm its therapeutic efficacy.

In the present study, tiamulin was administered directly into the crop. Vinothini et al. concluded that tiamulin had equal efficacy after administration in chickens by the three different routes (in-crop, in-water, and in-feed) as no significant difference in the pharmacokinetics parameters among the three routes was noticeable (16).

In conclusion, the results of this research suggested a dose of 35 mg/kg/day orally for tiamulin to fulfill the desired effect against *M. anatis* in ducks. Furthermore, a 6-day preslaughter withdrawal period is proposed following oral administration of tiamulin to assure safe human consumption of duck tissues.

## Data Availability Statement

The original contributions presented in the study are included in the article/supplementary materials, further inquiries can be directed to the corresponding author/s.

## Ethics Statement

The animal study was reviewed and approved by the Animal Ethics Committee at the Faculty of Veterinary Medicine, Mansoura University.

## Author Contributions

WH and SE designed the study, analyzed the data of this work, and wrote the manuscript. SE carried out the animal experiment. NE conducted the HPLC analysis. YH performed the PD studies including the *in vitro* and time-killing studies. S-CP did the daily dose calculations. All authors have read and approved the final manuscript.

## Conflict of Interest

The authors declare that the research was conducted in the absence of any commercial or financial relationships that could be construed as a potential conflict of interest.
